# Age-dependent differences in the association between blood interleukin-6 levels and mortality in patients with sepsis: a retrospective observational study

**DOI:** 10.1186/s40560-025-00775-1

**Published:** 2025-01-13

**Authors:** Takashi Shimazui, Takehiko Oami, Tadanaga Shimada, Keisuke Tomita, Taka-aki Nakada

**Affiliations:** Department of Emergency and Critical Care Medicine, Chiba University Graduate School of Medicine, 1-8-1 Inohana, Chiba, 260-8677 Japan

**Keywords:** Aged, Cytokines, Interleukin-6, Sepsis, Young adult

## Abstract

**Background:**

Interleukin-6 (IL-6) is a cytokine that predicts clinical outcomes in critically ill patients, including those with sepsis. Elderly patients have blunted and easily dysregulated host responses to infection, which may influence IL-6 kinetics and alter the association between IL-6 levels and clinical outcomes.

**Methods:**

This retrospective observational study included patients aged ≥ 16 years who were admitted to the intensive care unit at Chiba University Hospital. The patients were categorized into two groups: non-elderly (< 70 years) and elderly (≥ 70 years). Associations between log-transformed blood IL-6 levels and 28-day in-hospital mortality (primary outcome) and multiple organ dysfunction (MOD) on days 3 and 7 (secondary outcomes) were examined.

**Results:**

The non-elderly and elderly groups included 272 and 247 patients, respectively. There were no significant differences in the Sequential Organ Failure Assessment score, components of the APACHE II score (Acute physiology score and Chronic health points), MOD at baseline, or any of the outcome measures between the groups. In the non-elderly group, univariate Cox regression analysis showed a significant association between IL-6 levels and mortality (hazard ratio [HR] 1.71, 95% confidence interval [CI] 1.25–2.37, *P* < 0.001). This association remained significant after adjusting for sex, body mass index, steroid use prior to sepsis onset, and number of chronic organ dysfunctions (HR 1.66, 95% CI 1.20–2.32, *P* = 0.002). However, no significant association was observed in the elderly group in either the univariate (*P* = 0.69) or multivariable analyses (*P* = 0.77). Multivariable logistic regression analysis of MOD on days 3 and 7 revealed significant associations between MOD and IL-6 levels in both groups.

**Conclusions:**

Blood IL-6 levels were significantly associated with mortality in non-elderly patients with sepsis, but not in elderly patients. IL-6 levels were associated with MOD in both groups. Therefore, IL-6 levels should be interpreted with caution when predicting mortality in elderly patients with sepsis.

*Trial registration:* Not applicable.

**Supplementary Information:**

The online version contains supplementary material available at 10.1186/s40560-025-00775-1.

## Introduction

A dysregulated immune response to infection, provoked by humoral mediators, such as cytokines, is a key underlying mechanism of sepsis [1, 2]. Although an immune response is necessary to overcome infection, excessive levels of humoral mediators can trigger systemic inflammatory response syndrome (SIRS), leading to poor outcomes, such as death and multiple organ dysfunction (MOD) [3, 4]. Early recognition of exaggerated inflammatory responses is crucial for initiating timely treatment in patients at risk for poor outcomes, thereby improving their prognosis.

Interleukin-6 (IL-6) is a proinflammatory cytokine that accurately reflects the severity of the inflammatory response [5, 6]. Previous studies have demonstrated significant associations between elevated IL-6 levels and increased mortality or risk of MOD in patients with critical illness, including sepsis [6–8]. Based on these significant associations, IL-6 has been used as both a diagnostic and prognostic biomarker in critically ill patients [9, 10]. However, given that various factors such as aging are associated with immune system alterations [11, 12] that affect IL-6 expression, the associations between IL-6 levels and outcomes should be interpreted with caution, taking the patient’s background into account.

Elderly patients exhibit blunted and easily dysregulated host responses to infection, resulting in altered cytokine expression in sepsis compared to that in non-elderly patients [11, 12]. In addition, age-related chronic changes may lead to underlying inflammation, causing increased expression of cytokines, including IL-6, even in healthy elderly individuals [13]. These age-related differences may result in distinct IL-6 kinetics between elderly and non-elderly patients with sepsis, and could lead to different associations between IL-6 levels and clinical outcomes. Understanding these differences may enhance the accurate use of IL-6 as a biomarker, which can contribute to improving the clinical practice in sepsis. However, insufficient research has been conducted on these differences.

In this study, we hypothesized that the association between blood IL-6 levels and outcomes differs between non-elderly and elderly patients with sepsis. To elucidate these associations, we investigated the association between IL-6 levels and mortality or MOD across different age groups.

## Methods

### Study setting and patients

This single-center retrospective observational study investigated patients admitted to the medical/surgical intensive care unit (ICU) of Chiba University Hospital between October 2012 and December 2020. Of the 15,037 patients admitted to the ICU during the study period, we retrospectively screened 780 adult (≥ 16 years) patients suspected of having sepsis at the time of ICU admission. Of them, 755 patients were diagnosed with sepsis. Among them, IL-6 levels on ICU admission were measured in 519 patients who were enrolled in this study. All these patients were initiated on treatment for sepsis on the day of ICU admission. IL-6 levels were measured in patients suspected of being in the early phase of an inflammatory state, based on clinical judgment and necessity as determined by the physicians. Patients were excluded if they were aged < 16 years, did not satisfy sepsis criteria according to the Sepsis-3 criteria [1], had missing Sequential Organ Failure Assessment (SOFA) score data, or had not measured blood interleukin-6 levels on ICU admission. A scatter plot between age and IL-6 levels was illustrated to present a graphical overview of their correlation.

This study was approved by the Chiba University Hospital Certified Clinical Research Review Board (No. HK202402-01). As this was a retrospective observational study, the Review Board waived the need to obtain written informed consent in accordance with the Ethical Guidelines for Medical and Health Research Involving Human Subjects in Japan.

### Data collection and definition

Baseline characteristics, including age, sex, body mass index (BMI), chronic organ dysfunction (congestive heart failure, chronic lung disease, chronic kidney disease, chronic liver disease, and chronic central nervous system [CNS] disorder), steroid use prior to sepsis onset, septic shock, severity scores (Acute Physiology and Chronic Health Evaluation [APACHE] II score, components of the APACHE II score [Acute physiology score and Chronic health points], and SOFA score), and MOD at baseline were retrieved. Chronic organ dysfunctions were identified based on diagnoses recorded in the patient’s past medical histories retrieved from medical records. Cases with diagnoses indicative of CNS disorders underwent further review and were classified as chronic CNS disorders if they exhibited persistent consciousness disorders and/or paralysis. In-hospital 28-day mortality and MOD on days 3 and 7 were estimated as the outcome values. We chose these timepoints, because the recognition of MOD in the early phase is crucial for timely treatment [14]. All the outcome days were counted from the day of ICU admission.

Sepsis and septic shock were defined based on Sepsis-3 criteria [1]. Patients aged ≥ 70 years were defined as elderly and those with < 70 years were defined as non-elderly, according to the previous report indicating that patients aged ≥ 70 years have altered host responses to sepsis compared to the younger generation [11]. MOD was defined as more than or equal to 2 organs having SOFA score ≥ 2 [1, 15]. Patients were assumed to have MOD if they died before or on the day of estimation. Patients were assumed to not have MOD if they were alive and discharged before or on the estimation day [6].

### Statistical analysis

The primary outcome was the in-hospital 28-day mortality. Secondary outcomes were MOD on days 3 and 7. Cox regression or logistic regression analyses were performed to identify the associations between blood IL-6 levels and outcomes in different age groups. Based on the distribution in the studied patients (Supplementary Fig. 1) and supporting evidence from previous reports [16, 17], IL-6 levels were log-transformed to approximate a normal distribution for use in the analyses. Multivariable regression analyses adjusted for sex, BMI, steroid use prior to sepsis onset, and number of chronic organ dysfunctions were performed to adjust for baseline differences in both mortality and MOD. Covariates were selected, because they potentially impacted outcomes according to previous reports [18–22]. In addition, although severity scores have been reported to potentially associate with IL-6 levels [9, 23], and weak positive correlations between log-transformed IL-6 levels were confirmed in our patients (Supplementary Table 1), we further adjusted for the APACHE II score to evaluate the clinical importance of IL-6 in relation to mortality. Spearman’s rank correlation coefficient was used to evaluate the correlations between the variables. Subgroup analysis was performed using age categories divided by 10 years (< 50, 50–59, 60–69, 70–79, and ≥ 80 years) to identify the detailed association between IL-6 levels and mortality in different age groups. Differences in IL-6 levels between survivors and non-survivors were also analyzed in each group. The interaction term between age categories (non-elderly vs. elderly) and log-transformed IL-6 levels was tested using Cox regression analysis. The area under the receiver operating characteristic (ROC) curve (AUC) was used to evaluate the predictive value of IL-6 level for mortality. The Youden index for predicting mortality was used to determine the cutoff value of IL-6 between the high and low groups. A *Z* test was performed to compare the AUCs between the independent cohorts of the non-elderly and elderly groups. Log-rank tests between the high and low IL-6 groups were performed to determine differences in survival curves in each age category. The Pearson’s chi-square test was used to analyze categorical values, and the Wilcoxon test was used to analyze continuous values.

Data are expressed as medians (interquartile ranges [IQR]) for continuous variables and absolute numbers (%) for categorical variables. Statistical significance was set at *p* < 0.05. Analyses were performed using JMP Pro 15 software (SAS Institute Inc., Cary, NC, USA) or R version 4.4.2 with pROC package (R Foundation for Statistical Computing, Vienna, Austria).

## Results

Among the 519 enrolled patients, 272 non-elderly and 247 elderly patients formed the study cohort (Table 1, Supplementary Figs. 2,  3). Elderly patients had significantly lower BMIs than non-elderly patients (*P* = 0.018). No significant differences were found in chronic organ dysfunction, SOFA scores, or MOD at baseline between the groups. While elderly patients had significantly higher APACHE II scores (*P* = 0.015), there were no differences in Acute physiology scores and Chronic health points. Elderly patients had significantly higher blood IL-6 levels on admission than non-elderly patients (non-elderly vs. elderly, 1021 [244–11,404] vs. 1,767 [318–14,312] pg/mL, *P* = 0.029). A histogram of patients by IL-6 quintile illustrates that more non-elderly patients were in the lower quintiles, whereas elderly patients were more often in the higher quintiles (Supplementary Fig. 4). However, the scatter plot showed a minimal correlation between IL-6 levels and age (Supplementary Fig. 5). No significant differences were found in any of the outcome values between the groups (Table 1).

**Table 1:** Baseline characteristics and clinical outcomes

	Non-elderly (< 70 years) (*n* = 272)	Elderly (≥ 70 years) (*n* = 247)	*P* value
Characteristics			
Age, years	59 (46–66)	76 (73–81)	< 0.001
Male sex, n (%)	172 (63.2)	170 (68.8)	0.18
Interleukin-6, pg/mL	1,021 (244–11,404)	1,767 (318–14,312)	0.029
Septic shock, n (%)	73 (26.8)	71 (28.7)	0.63
Body mass index	23.1 (20.2–26.7)	22.5 (20.0–25.0)	0.018
Comorbidities n (%)			
Congestive heart failure	16 (5.9)	22 (8.9)	0.19
Chronic lung disease	11 (4.0)	19 (7.7)	0.075
Chronic kidney disease	27 (9.9)	23 (9.3)	0.81
Chronic liver disease	16 (5.9)	19 (7.7)	0.41
Chronic CNS disorder	13 (4.8)	12 (4.9)	0.97
Number of chronic organ dysfunction*	0 (0–1)	0 (0–1)	0.10
Steroid use prior to sepsis onset, n (%)	51 (18.8)	31 (12.6)	0.053
SOFA score	12 (9–16)	12 (9–15)	0.73
APACHE II score	29 (22–36)	31 (25–38)	0.015
Acute physiology score	24 (17–31)	23 (17–29)	0.27
Chronic health points	0 (0–5)	0 (0–5)	0.79
MOD at baseline, n (%)	216 (79.4)	198 (80.2)	0.83
Outcomes			
MOD on day 3, n (%)	217 (81.9)	193 (81.4)	0.90
MOD on day 7, n (%)	149 (66.2)	145 (71.4)	0.25
In-hospital 28-day mortality, n (%)	30 (11.0)	35 (14.2)	0.28

Median (interquartile range)

*CNS* central nervous system, *SOFA* Sequential Organ Failure Assessment, *APACHE* Acute Physiology and Chronic Health Evaluation, *MOD* multiple organ dysfunction

^*^Total number of congestive heart failure, chronic lung disease, chronic kidney disease, chronic liver disease, and chronic CNS disorders

*P* values were calculated using Pearson’s chi-square test and Wilcoxon test

### Associations between IL-6 levels and mortality across different age categories

**Fig. 1:**
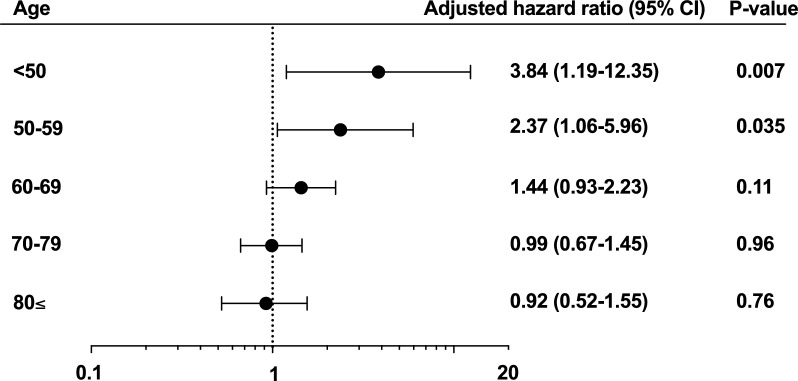
Multivariable Cox regression analysis to identify the associations between interleukin-6 and mortality in the detailed age categories. The adjusted hazard ratio was calculated using potential confounders, including sex, body mass index, steroid use prior to sepsis onset, and the number of chronic organ dysfunctions. The adjusted hazard ratio is associated with a one-unit change of log_10_ interleukin-6

In both the univariate and multivariable Cox regression analyses, higher IL-6 levels significantly increased the risk of death in the non-elderly patients (univariate for log_10_ IL-6, Hazard ratio [HR] 1.71, 95% confidence interval [CI] 1.25–2.37, *P* < 0.001; multivariable, HR 1.66, 95% CI 1.20–2.32, *P* = 0.002). However, there was no significant association between IL-6 and mortality in either univariate or multivariable analyses in elderly patients (univariate, *P* = 0.69; multivariable, *P* = 0.77) (Table 2). The different associations between IL-6 and mortality across age groups were also observed after adjusting for illness severity using the APACHE II score (Supplementary Table 2). Subgroup analysis in 10-year increments revealed a gradual decrease in the association between elevated IL-6 levels and mortality with patient age (Fig. 1). IL-6 levels were significantly higher in non-survivors among non-elderly patients (survivors vs. non-survivors, 866 [220–8,699] vs. 7,932 [546–114,825] pg/mL, *P* = 0.002), whereas no significant difference was observed in elderly patients (survivors vs. non-survivors, 1,765 [320–14,065] vs. 1,974 [252–14,414] pg/mL, *P* = 0.83). The interaction term between age categories and log-transformed IL-6 levels was statistically significant (*P* = 0.006).

**Table 2:** Cox regression analysis to identify the associations between interleukin-6 levels and mortality in the different age groups

	Non-elderly		Elderly	
	Hazard ratio (95% CI)	*P* value	Hazard ratio (95% CI)	*P* value
A. Univariate				
Log_10_ interleukin-6	1.71 (1.25–2.37)	< 0.001	0.94 (0.70–1.26)	0.69
B. Multivariable				
Log_10_ interleukin-6	1.66 (1.20–2.32)	0.002	1.05 (0.77–1.40)	0.77
Male sex	1.00 (0.47–2.20)	> 0.99	0.92 (0.46–1.93)	0.81
Body mass index	0.97 (0.90–1.04)	0.40	0.90 (0.81–0.99)	0.027
Steroid use prior to sepsis onset	1.12 (0.45–2.56)	0.79	3.75 (1.65–7.94)	0.002
Number of chronic organ dysfunction	1.93 (1.14–3.15)	0.016	1.26 (0.75–2.03)	0.37

Hazard ratio associated with a one-unit change of log_10_ interleukin-6

**Fig. 2:**
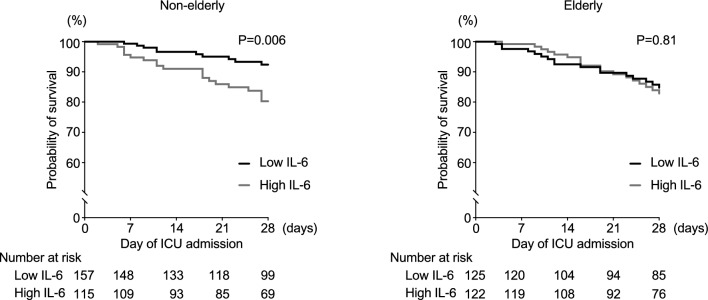
Probability of mortality in the groups categorized by the interleukin-6 levels. The cutoff value for Interleukin-6 levels was determined using the Youden index (1974 pg/mL)

The AUC value of IL-6 for mortality was significantly higher in non-elderly patients than in elderly patients (all patients, AUC 0.581 [cutoff value 1,974 pg/mL, sensitivity 58.5%; specificity, 56.2%]; non-elderly vs. elderly, AUC 0.675 vs. 0.512, *P* = 0.040). The survival curve in the high IL-6 group showed significantly higher mortality than that in the low IL-6 group among non-elderly patients (mortality, high IL-6 vs. low IL-6 group, 19.7% vs. 7.6%, *P* = 0.006). However, there was no difference between the high and low IL-6 groups in elderly patients (high IL-6 vs. low IL-6 group, 17.2% vs. 15.3%, *P* = 0.81) (Fig. 2).

### Associations between IL-6 levels and MOD across different age categories

In both the univariate and multivariable logistic regression analyses, and the non-elderly and elderly patients, higher IL-6 levels significantly increased the risk of MOD on day 3 (Supplementary Table 3). While univariate logistic regression analysis showed no significant association between IL-6 levels and MOD on day 7 in the elderly group (*P* = 0.090), multivariable logistic regression analyses demonstrated that higher IL-6 levels significantly increased the risk of MOD on day 7 in both the non-elderly and elderly groups (Supplementary Table 4).

## Discussion

Higher blood IL-6 levels upon ICU admission were significantly associated with an increased risk of death in non-elderly patients with sepsis. However, no such association was observed in the elderly patients. The significant interaction term between age categories and IL-6 levels further supported these different associations. In contrast, higher blood IL-6 levels were significantly associated with an increased risk of MOD on days 3 and 7 in both non-elderly and elderly patients.

Several studies have shown that higher IL-6 levels are associated with an increased risk of mortality in critically ill patients. A study with a small sample size (*n* = 142), including adult patients of all ages with sepsis or SIRS (median age [range], sepsis vs. SIRS, 75 [42–98] vs. 68 [37–81] years), suggested that increased serum IL-6 levels were associated with an increased risk of 28-day mortality (HR = 1 pg/mL change, 1.0004) [7]. A study including relatively younger critically ill patients (median age [IQR], 64 [51–74] years) indicated that plasma IL-6 levels > 100.9 pg/mL were associated with worse 90-day mortality (HR 1.91, 95% CI 1.62–2.24) and this association persisted even after adjustment for severity using Simplified Acute Physiology-II score (adjusted HR 1.92, 95% CI 1.63–2.26) [8]. Our results in non-elderly patients demonstrated that higher IL-6 levels were associated with an increased hazard ratio for in-hospital 28-day mortality, which is consistent with these reports.

The elderly patients in our study had significantly higher IL-6 levels at admission than the non-elderly patients. However, there was no significant association between IL-6 levels and risk of mortality in this group. Cytokine expression may differ between elderly and non-elderly individuals under septic conditions. A previous report on human sepsis suggested that patients aged ≥ 70 years showed decreased expression of genes involved in cytokine signaling and innate and adaptive immunity in blood leukocytes compared to patients aged < 50 years [11]. This finding indicates that elderly patients may have a blunted host response, which could contribute to their impaired cytokine expression during sepsis. Meanwhile, animal studies using lipopolysaccharide- or cecal ligation and puncture-induced sepsis showed that blood IL-6 levels were significantly higher in aged mice than in younger mice [24, 25]. This observation suggests that elderly animals may exhibit a more dysregulated host response compared to younger animals, even when subjected to the same insults. Given that the host responses in elderly patients may easily be dysregulated, the blurred cytokine expressions make their dynamics complex and not straightforward. Our study showed similar mortality rates between elderly patients with lower and higher IL-6 levels (Fig. 2). In addition, this outcome may be influenced by altered host responses; elderly patients exhibited high IL-6 levels even among survivors, whereas non-elderly survivors had low IL-6 levels, and non-elderly non-survivors displayed extremely high IL-6 levels (survivors vs. non-survivors: elderly, 1,765 vs. 1,974 pg/mL; non-elderly, 866 vs. 7,932 pg/mL). Age-related changes may influence cytokine dynamics and alter the association between IL-6 levels and mortality.

Unlike its association with mortality, higher blood IL-6 levels were significantly associated with an increased risk of MOD on days 3 and 7 in both the elderly and non-elderly patients. This finding highlights the potential clinical application of IL-6 as a biomarker even in elderly patients. IL-6 has been reported to be associated with organ dysfunction or severity of critical illness. Our previous study showed the blood IL-6 levels in the early phase of ICU admission (days 0, 1, 2) had high predictive values for MOD on days 3 and 7 among seven clinically relevant biomarkers (IL-6, IL-8, IL-10, tumor-necrosis-factor-α, white blood cells, C-reactive protein, and procalcitonin) [6]. A study involving critically ill patients including both with and without sepsis demonstrated that baseline plasma IL-6 levels were associated with the need for future organ support therapies, such as vasopressors/inotropes and renal replacement therapy [8]. In addition, an investigation that included critically ill patients of all ages who visited the emergency department demonstrated that IL-6 was significantly associated with a diagnosis of sepsis based on the sepsis-3 criteria, which is equal to the number of patients with organ dysfunction, with a high diagnostic value (AUC 0.764) [10]. Notably, this study found no significant association between IL-6 levels and 28-day mortality, which is consistent with the results in our elderly patients. Although several studies have reported that higher IL-6 levels are significantly associated with an increased risk of mortality [7, 8], this association remains controversial [26]. Our study demonstrated that higher IL-6 levels significantly increased the risk of MOD but showed no such association with the risk of mortality in elderly patients. Exaggerated inflammation in sepsis directly contributes to early phase deterioration. However, later-phase outcomes such as mortality may be influenced by factors other than inflammation, such as immune senescence. Therefore, the timing of the outcomes may partly explain the different associations in elderly patients. Moreover, non-elderly patients exhibit more robust immune responses and are capable of expressing cytokines more effectively than elderly patients [11], which may contribute to the significant association between IL-6 levels and the risk of mortality observed in non-elderly patients. IL-6 levels may serve as various outcome predictors in non-elderly patients, while it may be a more reliable indicator of the risk of MOD in the early phase rather than the risk of mortality in elderly patients.

Several covariates other than IL-6 levels were significantly associated with the risk of mortality in only one of the groups (non-elderly, number of chronic organ dysfunctions, *P* = 0.016; elderly, BMI and steroid use prior to sepsis onset, *P* = 0.027 and 0.002, respectively). Previous studies have suggested that these factors may affect outcomes differently across different age groups. Elderly patients with sepsis or septic shock had higher APACHE II scores than non-elderly patients, while they had similar numbers of chronic organ dysfunctions, suggesting that factors other than chronic organ dysfunction contribute more to severe conditions in elderly patients [27]. Overweight or obese BMI was associated with decreased mortality in patients with sepsis aged > 50, while it was not associated with mortality in those aged ≤ 50 years [20]. Although studies investigating the effects of steroid use before the onset of sepsis are limited, one study that included relatively younger patients with septic shock (median age, 68 years) found that baseline exposure to immunosuppressive therapy was not associated with mortality [28]. Age can be a crucial factor as it influences the association between covariates and outcomes.

Our study had several limitations. First, this was a single-center retrospective observational study, which may have caused selection, treatment, and misclassification biases. Although information such as comorbidities was retrieved using consistent criteria, some data may still be lacking. Furthermore, while we included more than 500 patients with sepsis, the generalizability of our findings should be investigated. Second, IL-6 levels were not measured based on pre-determined criteria, which may also have introduced selection bias. However, there was no significant difference in the proportion of the patients with missing IL-6 measurements between the non-elderly and elderly groups among those with sepsis (patients with missing IL-6 levels/patients with sepsis: non-elderly vs. elderly, 110/382 [28.8%] vs. 126/373 [33.8%], *P* = 0.14). Third, we could not determine the precise onset of sepsis, which may affect the timeline of MOD development. The occurrence and clinical course of MOD depend on the timing of the initial insult. Fourth, there is no universally established definition of age in elderly patients. Nonetheless, we conducted a subgroup analysis by dividing the patients into 10-year age groups and found a consistent trend of decreasing association between elevated IL-6 levels and mortality as age increased. This study found associations between IL-6 levels and mortality or MOD across different age groups. Despite these limitations, our findings provide valuable insights into IL-6 levels in patients with sepsis. However, further studies are required to establish a causal link between IL-6 levels and clinical outcomes.

## Conclusions

Higher blood IL-6 levels were significantly associated with an increased risk of mortality in non-elderly patients with sepsis; however, this association was not observed in elderly patients. Elevated IL-6 levels were significantly associated with an increased risk of MOD in the early phase in both non-elderly and elderly patients. Therefore, IL-6 levels should be interpreted with caution when predicting mortality in elderly patients with sepsis.

## Supplementary Information

Additional file 1.

Additional file 2.

Additional file 3.

Additional file 4.

Additional file 5.

Additional file 6.

## Data Availability

The data sets analyzed in the current study are available from the corresponding author upon reasonable request.

## Abbreviations

APACHE: Acute physiology and chronic health evaluation
AUC: Area under the receiver operating characteristic curve
BMI: Body mass index
CI: Confidence interval
CNS: Central nervous system
HR: Hazard ratio
ICU: Intensive care unit
IL-6: Interleukin-6
IQR: Interquartile range
MOD: Multiple organ dysfunction
OR: Odds ratio
ROC: Receiver operating characteristic curve
SIRS: Systemic inflammatory response syndrome
SOFA: Sequential organ failure assessment
