# Supplementary material for: Age-dependent differences in the association between blood interleukin-6 levels and mortality in patients with sepsis: a retrospective observational study
Source: J Intensive Care. 2025 Jan 13;13:3. doi: 10.1186/s40560-025-00775-1 (PMC11726927; doi:10.1186/s40560-025-00775-1)
Supplement: Supplementary file 1 — Additional file 1. [file 40560_2025_775_MOESM1_ESM.docx]

**Supplementary Fig. 1** Distribution of interleukin-6 levels in the studied patients.

A. Histogram for all patients with intervals of 50,000 pg/mL for interleukin-6 levels.

B. Histogram for patients with interleukin-6 levels below 5,000 pg/mL with intervals of 100 pg/mL for interleukin-6 levels.
