# Supplementary material for: Age-dependent differences in the association between blood interleukin-6 levels and mortality in patients with sepsis: a retrospective observational study
Source: J Intensive Care. 2025 Jan 13;13:3. doi: 10.1186/s40560-025-00775-1 (PMC11726927; doi:10.1186/s40560-025-00775-1)
Supplement: Supplementary file 2 — Additional file 2. [file 40560_2025_775_MOESM2_ESM.docx]

**Supplementary Fig. 2** Patient flow diagram.


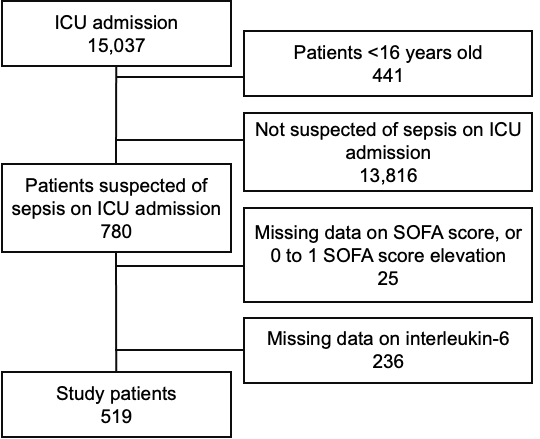


ICU, intensive care unit; SOFA, Sequential Organ Failure Assessment.
