# Supplementary material for: Age-dependent differences in the association between blood interleukin-6 levels and mortality in patients with sepsis: a retrospective observational study
Source: J Intensive Care. 2025 Jan 13;13:3. doi: 10.1186/s40560-025-00775-1 (PMC11726927; doi:10.1186/s40560-025-00775-1)
Supplement: Supplementary file 4 — Additional file 4. [file 40560_2025_775_MOESM4_ESM.docx]

**Supplementary Fig. 4** Interleukin-6 levels by quintiles in non-elderly and elderly patients.
