# Supplementary material for: Age-dependent differences in the association between blood interleukin-6 levels and mortality in patients with sepsis: a retrospective observational study
Source: J Intensive Care. 2025 Jan 13;13:3. doi: 10.1186/s40560-025-00775-1 (PMC11726927; doi:10.1186/s40560-025-00775-1)
Supplement: Supplementary file 6 — Additional file 6. [file 40560_2025_775_MOESM6_ESM.docx]

**Age-dependent differences in the association between blood interleukin-6 levels and mortality in patients with sepsis: a retrospective observational study**

Takashi Shimazui, Takehiko Oami, Tadanaga Shimada, Keisuke Tomita, Taka-aki Nakada

***Online data Supplement***

**Supplementary Table 1.** Spearman’s rank correlation coefficients between log-transformed interleukin-6 levels and severity scores in the studied patients.

|  | Correlation coefficient | P-value |
| --- | --- | --- |
| APACHE II score | 0.21 | <0.001 |
| SOFA score | 0.38 | <0.001 |

APACHE, Acute Physiology and Chronic Health Evaluation; SOFA, Sequential Organ Failure Assessment.

**Supplementary Table 2.** Multivariable Cox regression analysis further adjusted for severity score to identify the associations between interleukin-6 levels and mortality in the different age groups.

|  | Non-elderly | | Elderly | |
| --- | --- | --- | --- | --- |
|  | Adjusted odds ratio (95% CI) | P-value | Adjusted odds ratio (95% CI) | P-value |
| Log_10_ interleukin-6 | 1.54 (1.10-2.15) | 0.012 | 0.99 (0.73-1.34) | 0.94 |
| Male sex | 1.02 (0.48-2.18) | 0.96 | 0.82 (0.41-1.72) | 0.58 |
| Body mass index | 0.97 (0.91-1.04) | 0.43 | 0.84 (0.76-0.93) | <0.001 |
| Steroid use prior to sepsis onset | 1.11 (0.47-2.61) | 0.81 | 3.36 (1.40-7.56) | 0.008 |
| Number of chronic organ dysfunction | 1.86 (1.11-3.11) | 0.027 | 1.04 (0.62-1.66) | 0.86 |
| APACHE II score | 1.06 (1.01-1.10) | 0.010 | 1.12 (1.07-1.19) | <0.001 |

Hazard ratio associated with a one-unit change of log_10_ interleukin-6.

**Supplementary Table 3.** Logistic regression analysis to identify the associations between interleukin-6 levels and multiple organ dysfunction on day three in the different age groups.

A. Univariate

|  | Non-elderly | | Elderly | |
| --- | --- | --- | --- | --- |
|  | Odds ratio (95% CI) | P-value | Odds ratio (95% CI) | P-value |
| Log_10_ interleukin-6 | 1.75 (1.26-2.44) | <0.001 | 1.68 (1.20-2.35) | 0.001 |

B. Multivariable

|  | Non-elderly | | Elderly | |
| --- | --- | --- | --- | --- |
|  | Adjusted odds ratio (95% CI) | P-value | Adjusted odds ratio (95% CI) | P-value |
| Log_10_ interleukin-6 | 1.77 (1.27-2.48) | <0.001 | 1.74 (1.22-2.46) | 0.001 |
| Male sex | 1.60 (0.81-3.15) | 0.18 | 0.95 (0.45-2.04) | 0.90 |
| Body mass index | 1.02 (0.96-1.08) | 0.58 | 1.06 (0.97-1.16) | 0.18 |
| Steroid use prior to sepsis onset | 0.92 (0.37-2.26) | 0.86 | 1.10 (0.40-3.00) | 0.86 |
| Number of chronic organ dysfunction | 2.63 (1.11-6.24) | 0.013 | 1.21 (0.69-2.12) | 0.50 |

Odds ratio associated with a one-unit change of log_10_ interleukin-6.

**Supplementary Table 4.** Logistic regression analysis to identify the associations between interleukin-6 levels and multiple organ dysfunction on day 7 in the different age groups.

A. Univariate

|  | Non-elderly | | Elderly | |
| --- | --- | --- | --- | --- |
|  | Odds ratio (95% CI) | P-value | Odds ratio (95% CI) | P-value |
| Log_10_ interleukin-6 | 1.48 (1.13-1.93) | 0.003 | 1.26 (0.96-1.66) | 0.090 |

B. Multivariable

|  | Non-elderly | | Elderly | |
| --- | --- | --- | --- | --- |
|  | Adjusted odds ratio (95% CI) | P-value | Adjusted odds ratio (95% CI) | P-value |
| Log_10_ interleukin-6 | 1.53 (1.15-2.03) | 0.002 | 1.39 (1.03-1.87) | 0.026 |
| Male sex | 1.92 (1.03-3.58) | 0.040 | 0.89 (0.44-1.78) | 0.74 |
| Body mass index | 1.01 (0.96-1.07) | 0.62 | 1.02 (0.94-1.10) | 0.65 |
| Steroid use prior to sepsis onset | 1.20 (0.53-2.71) | 0.65 | 2.18 (0.75-6.33) | 0.12 |
| Number of chronic organ dysfunction | 2.24 (1.18-4.23) | 0.008 | 2.31 (1.24-4.29) | 0.004 |

Odds ratio associated with a one-unit change of log_10_ interleukin-6.
